# Transcatheter aortic valve replacement for pure aortic regurgitation: using left ventricular outflow tract implant as an auxiliary support

**DOI:** 10.1093/ehjcr/ytae185

**Published:** 2024-04-15

**Authors:** ChangDong Zhang, YuCheng Zhong, XiaoKe Shang, NianGuo Dong

**Affiliations:** Department of Cardiovascular Surgery, Union Hospital, Tongji Medical College, Huazhong University of Science and Technology, 1277 Jiefang Avenue, Jianghan District, 430022 Wuhan, China; Department of Cardiovascular Surgery, Union Hospital, Tongji Medical College, Huazhong University of Science and Technology, 1277 Jiefang Avenue, Jianghan District, 430022 Wuhan, China; Department of Cardiovascular Surgery, Union Hospital, Tongji Medical College, Huazhong University of Science and Technology, 1277 Jiefang Avenue, Jianghan District, 430022 Wuhan, China; Department of Cardiovascular Surgery, Union Hospital, Tongji Medical College, Huazhong University of Science and Technology, 1277 Jiefang Avenue, Jianghan District, 430022 Wuhan, China

**Figure:**
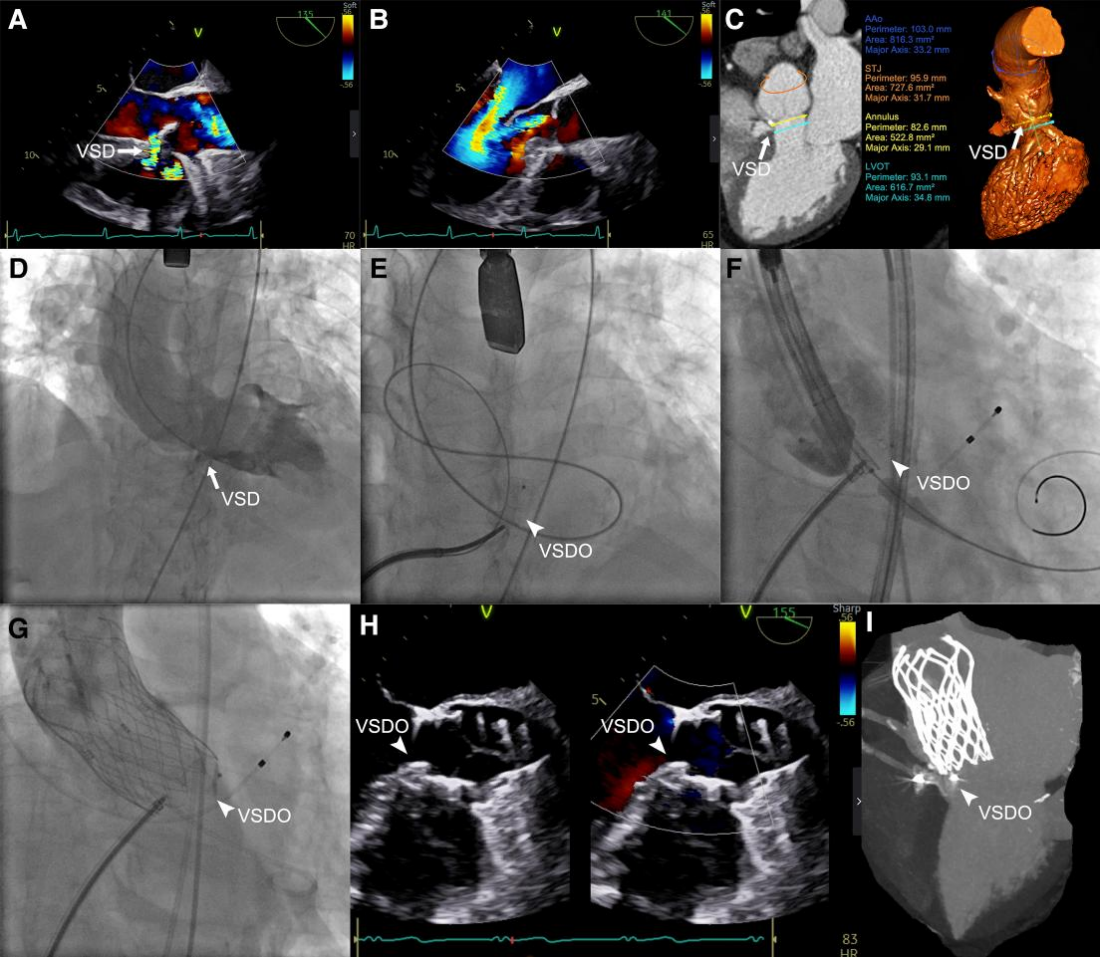


## Case description

A 73-year-old woman experienced post-exercising chest tightness and chest pain recurrently over the past decade. The echocardiography examination in our hospital showed that the patient suffered ventricular septal defect (VSD) (*Panel A*; the white arrow indicates VSD, the same below) and severe aortic valve insufficiency (*Panel B*), as well as secondary left ventricular dilation with systolic dysfunction. In order to identify the detailed anatomical features, we performed computer tomography angiography for the patient. The three-dimension reconstruction of computer tomography angiography illustrated the VSD located below the right coronary leaflet, while measured data of the left ventricular outflow tract, annulus, sinus duct junction, and ascending aorta diameters were 29.6, 28.4, 30.5, and 32.8 mm, respectively (*Panel C*). After team discussion, we decided to perform interventional operation for the patient. Left ventricular angiography was performed firstly (*Panel D*), and a 12 mm membrane VSD occluder (VSDO) (Beijing Starway Medical Technology Co., Ltd, China) was implanted (*Panel E*; white arrowhead indicates VSDO, the same below). The occluder was then used as a support at left ventricular outflow side (*Panel F*) for implanting a self-expanding transcatheter aortic valve (Shanghai MicroPort CardioFlow Medical Technology Co., Ltd, China) (*Panel G*). Intraoperative echocardiography showed that the valve stent was holding up the occluder (*Panel H*), and post-operative computer tomography angiography further confirmed this positional relationship without late displacement (*Panel I*). The echocardiography examination of 1-year follow-up visit showed satisfactory position and good function both for the occluder and the aortic interventional valve. To our knowledge, this is the first time that an implant in left ventricular outflow tract has been used as an auxiliary support for transcatheter aortic valve replacement, providing a new idea for the treatment of pure aortic valve regurgitation. It is noteworthy that this is not recommended for typical use, as it could entail future device failure (either the transcatheter aortic valve or the occluder).

## Data Availability

Data pertaining to this article are provided within the manuscript. Please contact the corresponding author to request further details.

## Notes

**Consent**: Informed consent was signed with compliance with the Committee on Publication Ethics (COPE) guidelines.

**Funding:** None declared.

